# High‐intensity exercise training using a rotarod instrument (RotaHIIT) significantly improves exercise capacity in mice

**DOI:** 10.14814/phy2.15997

**Published:** 2024-05-02

**Authors:** Jonathan J. Herrera, Christopher M. McAllister, Danielle Szczesniak, Rose‐Carmel Goddard, Sharlene M. Day

**Affiliations:** ^1^ Department of Molecular & Integrative Physiology University of Michigan Medical School Ann Arbor Michigan USA; ^2^ Medical Scientist Training Program University of Michigan Medical School Ann Arbor Michigan USA; ^3^ Department of Medicine, Division of Cardiovascular Medicine University of Pennsylvania Perelman School of Medicine Philadelphia Pennsylvania USA; ^4^ Department of Medicine, Division of Cardiovascular Medicine University of Michigan Medical School Ann Arbor Michigan USA

**Keywords:** exercise capacity, exercise training, mice, rotarod

## Abstract

Voluntary or forced exercise training in mice is used to assess functional capacity as well as potential disease‐modifying effects of exercise over a range of cardiovascular disease phenotypes. Compared to voluntary wheel running, forced exercise training enables precise control of exercise workload and volume, and results in superior changes in cardiovascular performance. However, the use of a shock grid with treadmill‐based training is associated with stress and risk of injury, and declining compliance with longer periods of training time for many mouse strains. With these limitations in mind, we designed a novel, high‐intensity interval training modality (HIIT) for mice that is carried out on a rotarod. Abbreviated as RotaHIIT, this protocol establishes interval workload intensities that are not time or resource intensive, maintains excellent training compliance over time, and results in improved exercise capacity independent of sex when measured by treadmill graded exercise testing (GXT) and rotarod specific acceleration and endurance testing. This protocol may therefore be useful and easily implemented for a broad range of research investigations. As RotaHIIT training was not associated cardiac structural or functional changes, or changes in oxidative capacity in cardiac or skeletal muscle tissue, further studies will be needed to define the physiological adaptations and molecular transducers that are driving the training effect of this exercise modality.

## 1 INTRODUCTION

The development and refinement of animal models of exercise (Feng et al., 2019; Hastings et al., 2022; Poole et al., 2020) has allowed researchers to interrogate the physiologic response to exercise training and the molecular transducers of physiological conditioning (Hawley et al., 2014; Vega et al., 2017). Several modalities have been developed for exercise testing of mice, including voluntary (e.g., wheel running) and forced (e.g., swim and treadmill‐based training) paradigms. Forced treadmill training is the most commonly used modality in animal models of exercise (Poole et al., 2020), as it enables precise control of the workload, including the intensity and duration of exercise training. Both moderate intensity endurance training and high‐intensity interval training (HIIT) can be performed on a treadmill (Hastings et al., 2022). HIIT typically affords a greater magnitude of a training effect, with marked improvements in the measurement of peak oxygen consumption (pVO_2_) achieved during graded exercise testing (GXT) (Hastings et al., 2022; Kemi et al., 2005; Ross et al., 2016; Wisloff et al., 2009).

However, treadmill‐based training has several disadvantages that can present significant challenges, be disruptive to, or confound an experiment. These include instrumentation expense and availability, risk of injuries (e.g., paw wounds, lacerations, and abrasions), use of noxious and/or stress provoking stimuli (e.g., electrical shock or air puffs) for motivation to achieve training compliance (i.e., the capacity to complete an exercise training session), and weekly pVO_2_ measurements to establish interval intensities (Kemi et al., 2002; Wisloff et al., 2001). Furthermore, the burst pattern of running on a treadmill can decrease the total amount of exercise training that an animal will perform willingly (Poole et al., 2020).

To address some of the drawbacks of treadmill training, we developed a HIIT protocol adapted for use on a rotarod‐a rotating rod with adaptable speed. The HIIT protocol was modeled after clinical cardiac rehabilitation programs which achieved improvements in pVO_2_ (Abad et al., 2017; Ito, 2019) integrated components from other murine interval training protocols (Kemi et al., 2002; Wisloff et al., 2001), and accounted for exercise compliance previously documented in C57BL/6J mice (Gibb et al., 2016). Using this novel training modality that we have called RotaHIIT, we demonstrate a significant training effect in female and male middle‐aged C57BL/6J mice as measured by GXT, the gold standard for evaluating cardiorespiratory fitness (Petrosino et al., 2016). GXT, however, requires costly equipment, trained personnel, and is not universally available to investigators (Poole et al., 2020). In this study, we further evaluate exercise performance using acceleration and endurance testing specific to the rotarod device. RotaHIIT provides a training modality that overcomes many of the limitations of treadmill training and may be used as an alternative to other forms of exercise training across a spectrum of mouse models of cardiovascular disease.

## 2 METHODS

### 2.1 Animals

Female and male C57BL/6J wild‐type mice were purchased from The Jackson Labs (Bar Harbor, ME). Breeder mice were provided LabDiet 5008 mouse feed, and weaned mice were fed LabDiet 5L0D (Lab Diet, Oxford, MI). Mice were group‐housed in plastic cages with metal tops and air‐filter vent cage tops. The cages were supplied with corn‐cob bedding (Bed O'Cobs, Andersons Lab Bedding, Maumee, OH). Ad libitum access to water was provided to mice in bottles. Mice in colony were housed in ventilated cages that were changed weekly. Housing room temperature was maintained within a range of 21–23°C. All protocols were submitted to, and approved, by the University of Michigan Institutional Animal Care and Use Committee.

### 2.2 Mouse cohorts and group numbers

A single cohort of middle aged (14.0 month ± 1.85) mice was used for all the experimental procedures with the exception of echocardiography (see below). Group numbers included the following: female: sedentary (*n* = 7), RotaHIIT (*n* = 7); male: sedentary (*n* = 10), RotaHIIT (*n* = 9). Animals were randomized to treatment group (e.g., RotaHIIT) or sedentary conditions.

For echocardiography, post echocardiographic imaging for five of the sedentary female mice and three of the sedentary males in the above cohort utilized for all other experimental measures were unable to be obtained due to logistics with the echocardiography core during the COVID‐19 pandemic. Therefore, six separate age‐ and sex‐matched female sedentary mice were used for echocardiography measures, but no additional sedentary male mice were added as numbers in this group were well matched to other groups. *Group numbers for echocardiography included the following*: female: sedentary (*n* = 8), RotaHIIT (*n* = 7); male: sedentary (*n* = 7), RotaHIIT (*n* = 9).

### 2.3 RotaHIIT training

**FIGURE 1:**
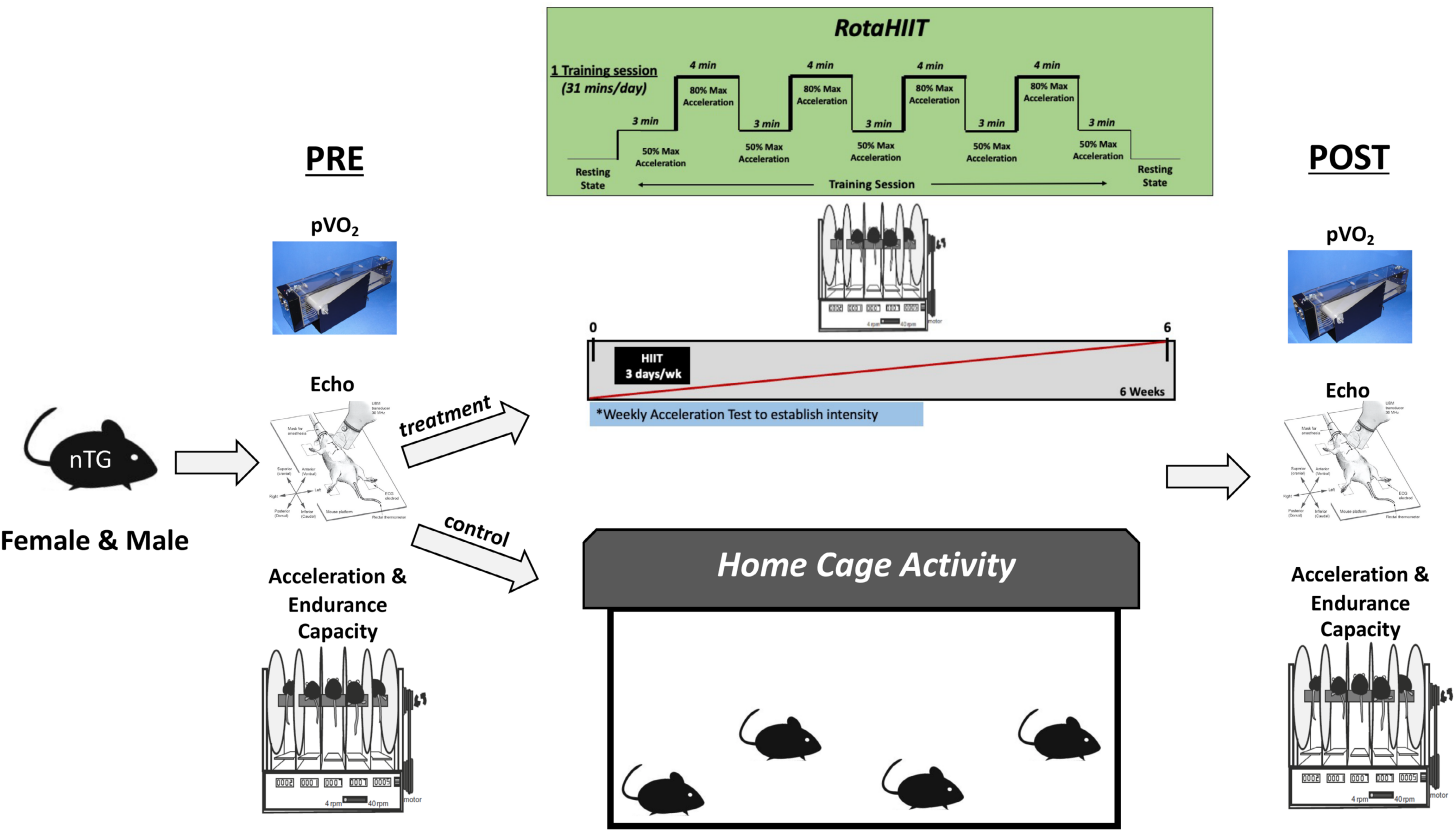
Rotarod high‐intensity interval training (RotaHIIT) experimental design and methodology. RotaHIIT employs a rotarod as the apparatus for forced exercise training. Prior to (pre) and following (post) training, mice were subjected to graded exercise testing, echocardiography and rotarod specific exercise capacity testing. For training, mice were subjected to a total training period of 6 weeks (with an additional week preceding the training period for rotarod acclimation). Three days per week, mice participated in a single training session that lasted 31 min and included a warm up (3 min), as well as low‐intensity (4 × 4 min) and high‐intensity (4 × 4 min) intervals. Interval intensities were determined by measuring group average maximum acceleration prior to start of each week, which increased every week (represented by red diagonal line; see Figure 2 for specific weekly interval intensities). Mice exercised at 50% and 80% of group average maximum acceleration achieved for low and high‐intensity intervals, respectively (see Figure 2 for corresponding rotarod RPMs).

**FIGURE 2:**
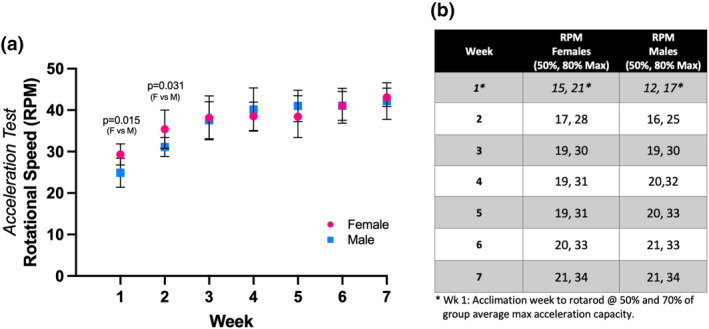
Average weekly maximum acceleration and established training workloads for RotaHIIT. Prior to the start of each week of exercise training, mice were subjected to a rotarod acceleration capacity test to establish RotaHIIT workloads. (a) Average maximum acceleration (± standard error of mean) for female and male mice are plotted for each week. (b) RotaHIIT workload intensity was determined by taking a percentage of the group average maximum acceleration capacity [i.e., maximum speed (revolutions per minute or RPM) achieved on rotarod]. RotaHIIT low‐intensity intervals were 50% and high‐intensity intervals were 80% of group average maximum acceleration capacity. (*Week 1 was utilized an as acclimation period for mice to become accustomed to rotarod). Unpaired *t*‐test was used to compare female and male averages per week (alpha threshold, *p* < 0.05).

Rotarod high‐intensity interval training (RotaHIIT) was conducted at the University of Michigan Physiology Phenotyping Core (UM PPC) (Figures 1 and 2 provide training protocol details). All training occurred between the hours of 8:00 a.m. and 12:00 p.m. (during beginning hours of sleep cycle), and was carried out on a Rat Mouse Rotarod (IITC Life Science, Woodland Hills, CA). The rotarod was specifically set up to run mice for this experiment, given that the rotarod is capable of accommodating rats or mice. The rotarod rod diameter measures at 0.032 m, and produces rotational speed in rotations per minute (RPM). For linear velocity conversion, the following formula was used: velocity (*v*) = radius (*r*) × RPM × 0.10472. Before each individual training session, the rotarod was thoroughly cleaned with 70% ethanol. Mice were trained for a total period of 7 weeks, with the first week serving as an acclimation period. One day prior to the start of each training week, mice were put through an acceleration test to establish the workload intensity for each training week (see *Adjunct Exercise Capacity* method section below for specific details as to how the acceleration tests were conducted). Each training week included three total training days, during which mice were subjected to a single RotaHIIT training session that lasted 31 min (1860 s). A single day training session included a 3‐min warm up at 50% of group maximum acceleration achieved during weekly acceleration test, as well as four low‐intensity (3 min each) and four high‐intensity intervals (4 min each). The weekly workload intensity was set to 50% and 80% of the group maximum acceleration achieved during an acceleration test each week for low‐ and high‐intensity intervals, respectively (Figure 2 provides specific RPMs used during each training week). To track training compliance, or the capacity for the mice to complete a training session, 5 s were subtracted from each mouse's total running time each time they fell off the rotarod during a single training session. This was determined to be the time necessary to gather and place a mouse back onto the rotating rod. The fall distance was estimated to be 1–1.5 inches. Long forceps were used to minimally assist mice and restore transient imbalance that occurred during training. Compliance was calculated as the total average training session run time per group for each training week.

### 2.4 Peak oxygen consumption and maximum treadmill speed

Peak oxygen consumption (pVO_2_) was measured at the University of Michigan Metabolic Mouse Phenotyping Center (UM MMPC). All tests were randomized, blinded, and conducted by trained technicians. Measurements were made using the Comprehensive Laboratory Animal Monitoring System (CLAMS, Columbus Instruments), an integrated open‐circuit calorimeter. Prior to pVO_2_ testing, mice were each placed into sealed treadmill chambers (305 × 51 × 44 mm^3^) for 10 min for treadmill environment acclimation with the motor on but without the track moving. Two days prior to the experimental testing, mice were placed onto the same treadmill for 30 min per day for additional acclimation. On the experiment test day, mice were individually placed into the sealed treadmill chambers and underwent a murine graded exercise test (GXT), adapted from Petrosino et al. (2016). The treadmill was positioned with no incline for the first 2 min of running, was set at 5° incline for next 2 min, then set to 10° incline for the next 2 min, and finally set at 15° incline for the remainder of the test. Exhaustion was determined when a mouse sat on the shocker (1.60 mA, 120v, 3 Hz) for five consecutive seconds, at which point the shocker was shut off, the treadmill stopped, and 10 min of recovery data recorded. Treadmill acclimation and the murine GXT were carried out in an experimentation room set at 20–23°C. Measurements were carried out between 9:00 a.m. and 14:00 p.m. (beginning hours of sleep cycle) on each day. The treadmill was wiped clean with 70% ethanol between each test. The system was routinely calibrated before the experiment using a standard gas (20.6% O_2_ and 0.5% CO_2_ in N_2_). VO_2_ and VCO_2_ in each chamber were sampled continuously every 5 s. The air flow rate through the chambers was set at 0.80 LPM. Maximal treadmill speed was recorded for each animal during the GXT.

### 2.5 Adjunct exercise capacity

Rotarod acceleration and endurance capacity testing were conducted at the University of Michigan Physiology Phenotyping Core (UM PPC). Both functional tests were conducted in a single, 1‐week period and included a rest day between each test for each mouse. All testing was randomized and blinded, and conducted between 8:00 a.m. and 12:00 p.m. (beginning hours of sleep cycle). Rotarod testing was performed using a Rat Mouse Rotarod (IITC Life Science, Woodland Hills, CA). For the acceleration capacity test, an initial speed of 5 RPM was set and the rotarod was then accelerated at 0.1 RPM/s. Fall latency, or the time mice were able to remain on the rotating rod prior to falling off the rod (i.e., a greater fall latency indicates that a mouse was able to stay on the rotating rod), was recorded over three trials with 1‐min rest between trials. Average fall latency over the three trials was used for analysis. For endurance capacity testing, the initial rotarod speed was set at 5 RPM, and then accelerated at 0.1 RPM/s over 15 min until the speed reached the group‐specific average speed achieved during acceleration testing (31 RPM). Mice could then continue for an additional 15 min, providing a total potential run time of 30 min. Mice were allowed up to 10 falls within the first 15 min and up to two falls in the remaining 15 min. Fall latency was scored once the mouse fell more than the permitted criteria. For both acceleration and endurance capacity tests, the rotarod was cleaned with 70% ethanol between each series of mice that were tested. Up to five mice could be tested at one time.

### 2.6 Echocardiography

Echocardiography was conducted at the University of Michigan Physiology Phenotyping Core (UM PPC) by a single, trained sonographer who was blinded to genotype and treatment group. Induction of anesthesia was performed in an enclosed container filled with 5% isoflurane. Following induction, mice were then placed on a warming pad to maintain body temperature. A total of 1%–1.5% isoflurane was provided through a nose cone to maintain a surgical plane of anesthesia. Mouse hair was removed from the upper abdominal and thoracic area with depilatory cream. ECG was monitored via noninvasive resting ECG electrodes. Transthoracic echocardiography was performed in the supine or left lateral position. Two‐dimensional (B‐mode), M‐mode, pulsed wave Doppler, and tissue Doppler images were recorded using a Visual Sonics' Vevo 2100 high‐resolution in vivo microimaging system.

### 2.7 Body weight and body composition

Mice were weighed prior to body composition analysis. Body fat, lean mass, free water, and total water were then measured using an NMR‐based analyzer‐EchoMRI, 4in1‐500 (Bruker. Allenstown, PA). Mice were individually placed in a measure tube, and remained for the measurement of ~2 min. NMR‐based analyzer is checked and calibrated daily using a reference sample (canola oil) as recommended by the manufacturer.

### 2.8 Western blotting

Western blot was performed on protein lysates prepared from the left ventricle and gastrocnemius muscle of sedentary and RotaHIITmice (*n* = 7–10). Protein was isolated in 1x Bio‐Rad laemmli buffer (Bio‐Rad laboratories, Richmond California, cat. 1610747) containing cOmplete mini (Millipore Sigma, Milwaukee Wisconsin, cat. 11836153001), and PhosSTOP (Millipore Sigma, Milwaukee Wisconsin, cat. 4906845001). Protein concentration was determined using the Bio‐Rad DC protein assay (Bio‐Rad laboratories, Richmond California, cat. 5000111). 40 μg of protein per sample was separated on a 4%–20% graded Invitrogen tris‐glycine plus gel (Thermo Fisher, Rockford Illinois, cat. wxp42020box) and wet‐transferred to 0.45 μm nitrocellulose membrane (Bio‐Rad laboratories, Richmond California, cat. 1620115). The total OXPHOS rodent primary antibody cocktail (Abcam, Boston Massachusetts, cat. ab110413) was used to stain oxidative phosphorylation complexes I–V (1:1000). GAPDH staining was achieved using a rabbit anti‐GAPDH primary antibody (Millipore Sigma, Milwaukee Wisconsin, cat. ABS16) (1:1000) and TFAM staining was performed using the rabbit anti‐TFAM primary antibody (Millipore Sigma, Milwaukee Wisconsin, cat. AV36993) (1:500). Li‐COR 680RD goat anti‐mouse (cat: 926‐68070) or goat‐anti rabbit (Li‐cor Biosciences, Lincoln Nebraska, cat: 926‐68071) IgG secondary antibodies were used (1:10,000). LI‐COR Odyssey Fc was used to visualize the signal and quantification was performed using Image Studio (version 5.2).

### 2.9 Statistics

Three statistical approaches were used in this study. (1) To compare weekly maximum acceleration and weekly compliance averages between female and male mice, we used unpaired *t*‐tests with an alpha threshold of *p* < 0.05. (2) To compare differences between groups (i.e., sedentary vs. RotaHIIT and male vs. female) in the change in peak oxygen consumption (pVO_2_), acceleration and endurance capacity, and echocardiography parameters over time, a one‐way ANOVA with Tukey's post hoc tests determined significance (alpha threshold, *p* < 0.05). (3) For the same experimental measures (pVO_2_, maximal treadmill speed, acceleration capacity, and endurance capacity), we used a repeated measures *t*‐tests to assess pre and post mean differences within a group in both sexes. A Pearson correlation analysis was conducted to evaluate the relationship between pVO_2_ and functional capacity (alpha threshold, *p* < 0.05).

## 3 RESULTS

### 3.1 Establishing training workload intensity with weekly maximum acceleration testing

In order to maximize training effects, exercise intensity needs to increase over time to account for training adaptations (Kemi et al., 2002). We measured average weekly maximum acceleration on the rotarod in order to set workload intensities for the RotaHIIT intervals as the exercise training progressed over time. In both female and male mice, maximum acceleration increased each week over the duration of training (Figure 2). After 7 weeks, female RotaHIIT‐trained mice reached an average maximum acceleration capacity of 42.97 ± 2.25 RPM, corresponding to a 46.6% increase compared to their initial average maximum acceleration capacity (Week 1: 29.31 ± 2.54 RPM). Male RotaHIIT‐trained mice reached an average maximum acceleration capacity of 42.20 ± 4.43 RPM, or a 69.48% increase from the initial average maximum acceleration capacity (Week 1: 24.92 ± 3.51 RPM). Average maximum acceleration was lower in male RotaHIIT‐trained mice during Week 1 (*p* = 0.015) and Week 2 (*p* = 0.031) when compared to female counterparts; however, these sex differences dissipated over the duration of training. Rotational speed (in RPMs) converted to linear velocity (in m/min) is displayed in Figure S1.

### 3.2 Compliance was high in both female and male mice

**FIGURE 3:**
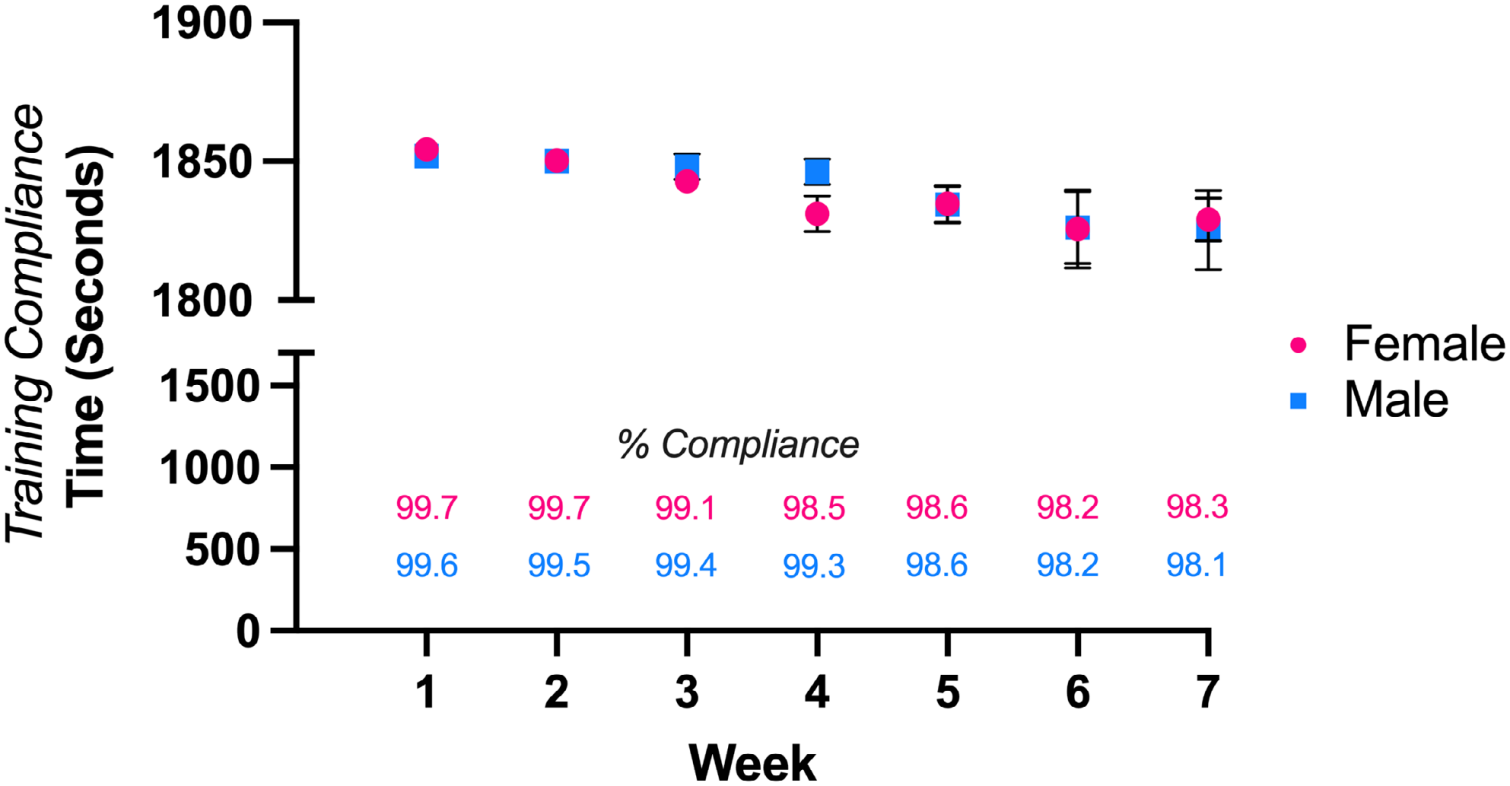
RotaHIIT training compliance. In a single training session, each mouse was subjected to exercise for 1860 s (31 min). During the training session, 5 s was subtracted from the total time for each fall off the rotating rod (i.e., time necessary to gather and place mouse back on rod). Compliance, or the total average training session run time per group (i.e., females or males) per week is plotted (± standard error of mean). Percentage of total possible running time achieved is listed at bottom of figure for each sex. Unpaired *t*‐tests were used to compare weekly compliance between female and male mice (alpha threshold, *p* < 0.05).

Exercise compliance can be highly variable across animal strains and across the different training modalities that are employed in murine exercise studies (Gibb et al., 2016). Notably, C57BL/6J mice are typically poorly compliant treadmill runners but highly compliant voluntary free wheel runners (Lerman et al., 2002; Massett & Berk, 2005). To measure compliance using the RotaHIIT protocol, we tabulated the total running time, or the total time the mouse remained on the rotating rod, for each individual mouse. Both female and male mice completed greater than 99% of the total training time through the first 3 weeks of training, and completed greater than 98% each of the last 3 weeks of training (Figure 3). Additionally, female and male mice did not differ in training compliance during any week of training.

### 3.3 RotaHIIT leads to increased peak oxygen consumption, rotarod acceleration, and endurance capacity in female and male mice without structural or functional changes measured by echocardiography

**FIGURE 4:**
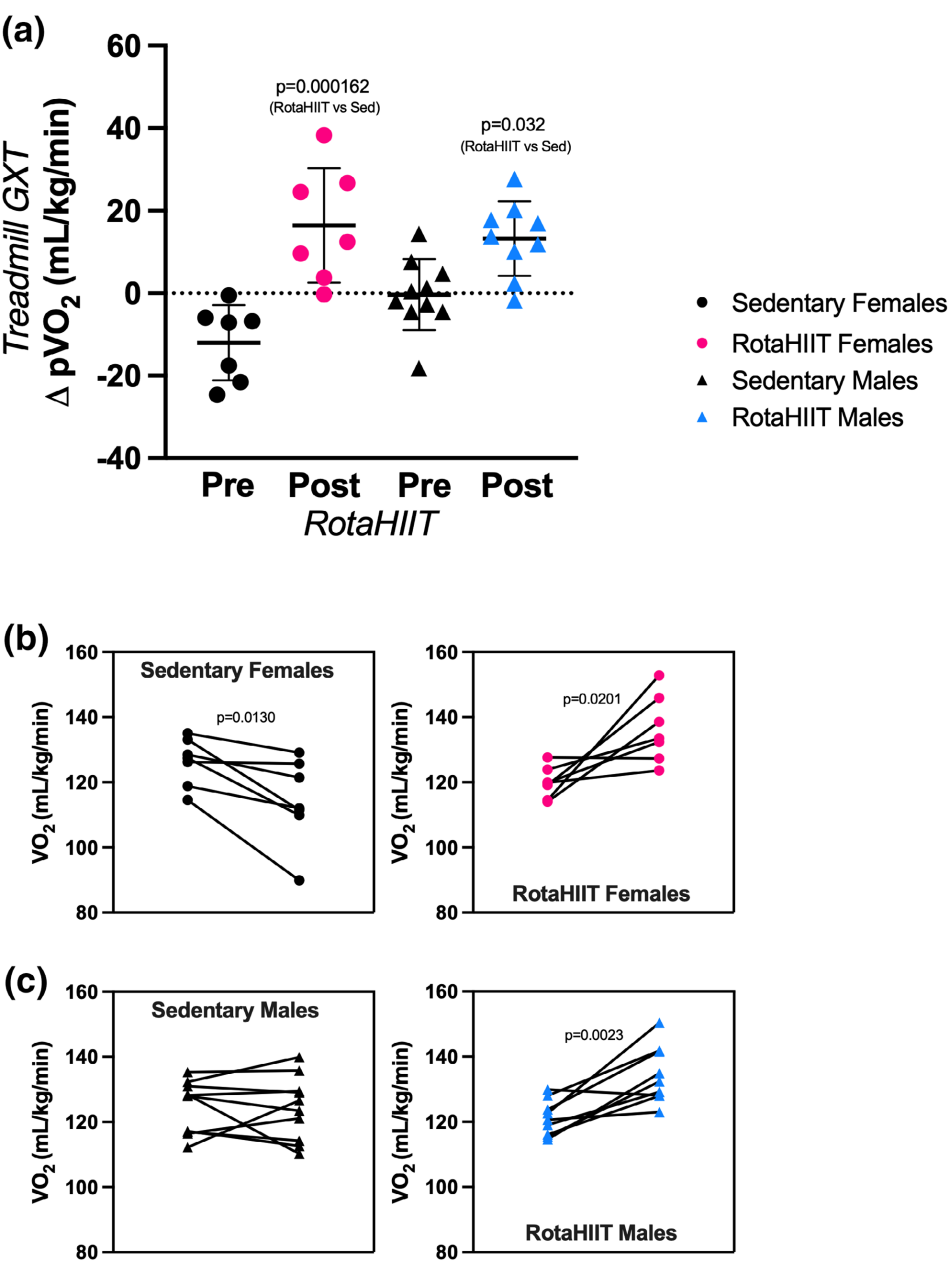
Change in peak oxygen consumption (pVO_2_). Peak oxygen consumption (pVO_2_) testing was carried out prior to (pre) and following (post) 6 weeks of RotaHIIT training in female and male mice. (a) Change in maximal oxygen consumption (Δ pVO_2_) was measured in a modular metabolic treadmill using a graded exercise test (GXT). Each plotted value represents post intervention average minus pre intervention average for a single mouse (± standard error of mean). (b, c) Pre and post pVO_2_ in the same cohort of mice. pVO_2_ was normalized to mouse body weight measured on day of GXT. One‐way ANOVA was used to compare changes across groups. Tukey's post hoc test was used to determine individual comparisons (i.e., sedentary vs. RotaHIIT) with alpha threshold of *p* < 0.05. Repeated measures *t*‐test was performed to assess mean differences within each group (i.e., pre vs. post) with alpha threshold of *p* < 0.05.

**FIGURE 5:**
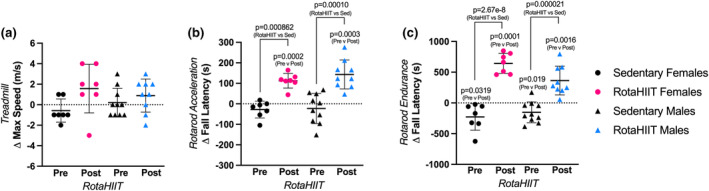
Change in maximal treadmill speed and rotarod exercise capacity testing. Prior to and following the 6‐week RotaHIIT intervention period, maximal speed achieved on a treadmill was measured, as well as acceleration and endurance capacity on the rotarod as adjuncts to peak oxygen consumption (pVO_2_) testing. (a) Average change in maximal treadmill speeds achieved at a 15° incline as measured by velocity during graded exercise testing (GXT) (± standard error of mean). Average change in rotarod acceleration (b) and endurance capacity (c) as measured by fall latency or the time a mouse remained on the rotating rod (± standard error of mean). One‐way ANOVA was used to compare changes across groups. Tukey's post hoc test was used to determine individual comparisons (i.e., sedentary vs. RotaHIIT) with alpha threshold of *p* < 0.05. Repeated measures *t*‐test was performed to assess mean differences within each group (i.e., pre vs. post) with alpha threshold of *p* < 0.05.

**FIGURE 6:**
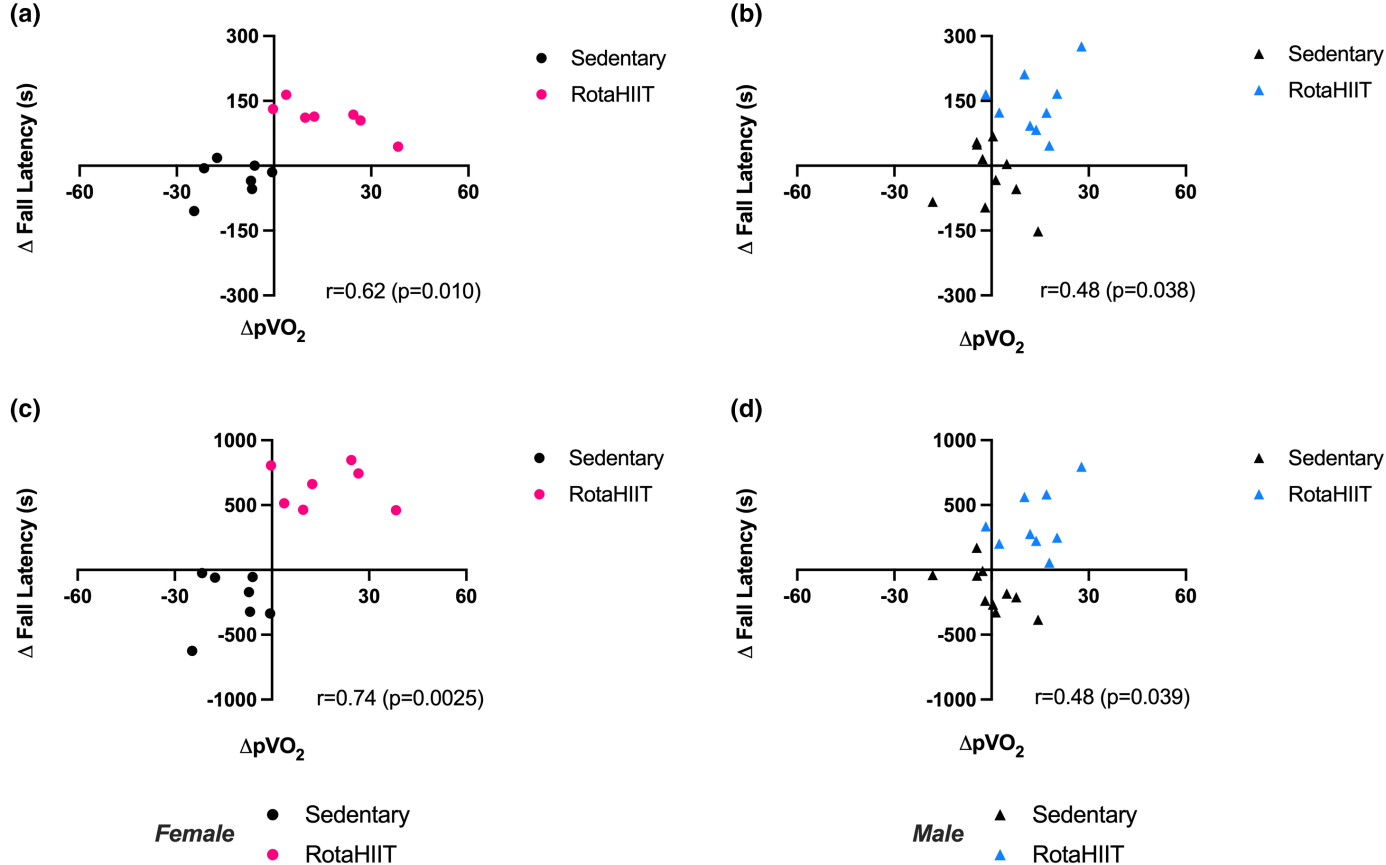
Correlation of pVO_2_ and functional capacity on the rotarod. (a, b) Change in pVO_2_ versus change in acceleration capacity in female and male mice. (c, d) Change in pVO_2_ versus change in endurance capacity in female and male mice. Pearson correlation was used to determine linear relationship between variables with alpha threshold of *p* < 0.05 for statistical significance.

Graded exercise testing (GXT) is the standard for evaluating respiratory capacity, that is, cardiovascular fitness. (Swain & American College of Sports Medicine, 2014). Accordingly, we measured pVO_2_ using a murine treadmill GXT (Petrosino et al., 2016) prior to and following the RotaHIIT intervention period. There were no baseline (i.e., pre‐intervention) differences in pVO_2_ between groups. RotaHIIT led to a significant increase in pVO_2_ in both female (+13.7% increase, *p* = 0.0201) and male (+10.9%, *p* = 0.0023) mice (Figure 4) compared to pre‐training baseline. Sedentary female mice had a decrement in pVO_2_ following the intervention period (−9.52%, *p* = 0.0130) while sedentary male mice demonstrated no significant change. The between‐group difference in pVO_2_ was significant between female and male RotaHIIT‐trained mice when compared to sedentary mice (female‐sedentary vs. RotaHIIT: *p* < 0.001; male‐sedentary vs. RotaHIIT: *p* = 0.032). There were no differences in respiratory exchange ratio (RER) between groups following the RotaHIIT intervention period (Figure S2) when pVO_2_ was achieved. RotaHIIT‐trained mice also demonstrated improvements compared to baseline in both rotarod acceleration (female: +57.7%, *p* = 0.0002; male: +90.5%, *p* = 0.0003) and endurance capacity (female: +67.2%, *p* = 0.0001; male: +38.4% *p* = 0.0016, Figure 5). Significant between‐group differences for change in acceleration capacity and endurance capacity on the rotarod were also observed in female and male mice compared to mice that remained sedentary (female and male‐sedentary vs. RotaHIIT: *p* < 0.001 for both acceleration and endurance capacity, Figure 5). Maximal rotational speed achieved on the rotarod was significantly faster in RotaHIIT trained compared to sedentary mice independent of sex (RotaHIIT vs female and male‐sedentary: *p* < 0.001, Figure S3). Maximal total distance, achieved during the rotarod endurance capacity test, was also longer in RotaHIIT trained mice (RotaHIIT vs female and male‐sedentary: *p* < 0.001, Figure S3). There were no significant differences within groups or in the change in treadmill maximal speed when pVO_2_ was achieved (Figure 5). The changes in acceleration and endurance capacity were positively correlated with the change in pVO_2_ in both sexes (Figure 6). Echocardiography did not reveal any significant differences between or within groups in cardiac morphology or function following RotaHIIT training in either males or females compared to sedentary controls (Table 1, Supplemental data file). Further analysis of postmortem gross heart weights normalized to body weight did not reveal any differences between RotaHIIT trained and sedentary mice independent of sex (Figure S4).

**TABLE 1:** RotaHIIT echocardiography data.

	Sedentary female		RotaHIIT female		Sedentary male		RotaHIIT male	
	Pre	Post	Pre	Post	Pre	Post	Pre	Post
HR (BPM)	535 ± 11.8	559 ± 10.7	533 ± 9.40	511.93 ± 7.84	507 ± 24.6	500 ± 16.3	498 ± 15.2	466 ± 21.0
LVDd (mm)‐M	3.92 ± 0.160	3.86 ± 0.134	3.69 ± 0.060	3.68 ± 0.071	4.19 ± 0.127	4.26 ± 0.168	3.93 ± 0.127	3.85 ± 0.127
LVDs (mm)‐M	2.88 ± 0.130	2.89 ± 0.117	2.68 ± 0.076	2.70 ± 0.076	3.33 ± 0.136	3.18 ± 0.168	2.95 ± 0.099	2.86 ± 0.156
LV PWd (mm)‐M	0.753 ± 0.53	0.769 ± 0.040	0.755 ± 0.021	**0.770 ± 0.024**	0.762 ± 0.021	0.764 ± 0.062	0.763 ± 0.029	0.804 ± 0.037
LV PWs (mm)‐M	1.13 ± 0.052	1.10 ± 0.063	1.09 ± 0.039	1.09 ± 0.026	1.01 ± 0.025	1.12 ± 0.069	1.10 ± 0.034	1.11 ± 0.029
LV Mass (mg)‐M	113 ± 15.4	116 ± 11.7	98.1 ± 3.11	101.47 ± 4.33	122.9 ± 10.2	129.9 ± 8.4	112 ± 7.73	118 ± 8.98
LV VOLd (μL)‐M	68.1 ± 6.78	65.1 ± 5.47	58.0 ± 2.22	58.0 ± 2.62	79.0 ± 5.98	82.6 ± 7.75	68.0 ± 5.49	64.9 ± 5.39
LV VOLs (μL)‐M	32.4 ± 3.62	32.6 ± 3.03	27.1 ± 1.88	27.7 ± 1.85	45.9 ± 4.81	41.5 ± 5.50	34.3 ± 2.84	32.3 ± 4.64
CO‐B	18.3 ± 1.17	17.0 ± 1.13	15.8 ± 0.643	14.1 ± 0.636	15.8 ± 0.852	18.1 ± 0.725	15.1 ± 1.23	17.1 ± 2.71
EF%‐B	54.8 ± 0.88	51.2 ± 2.37	52.9 ± 1.35	52.7 ± 1.37	48.2 ± 1.90	50.0 ± 0.021	48.2 ± 2.10	50.0 ± 2.68
FS%‐B	19.0 ± 1.94	17.8 ± 1.43	17.5 ± 1.24	16.8 ± 1.27	14.4 ± 0.029	14.2 ± 0.028	18.0 ± 1.73	16.3 ± 1.23
SV‐B	34.4 ± 2.52	30.6 ± 2.07	29.7 ± 1.23	27.7 ± 1.33	31.3 ± 1.18	36.5 ± 2.22	30.1 ± 2.01	31.7 ± 1.47

*Note*: Pre and post values ± standard error of mean (SEM) for each echocardiography measure is provided per group. Statistical analysis included (1) one‐way ANOVA comparing change (post minus pre) across groups, and (2) paired *t*‐tests comparing pre and post data within each group (statistical significance with alpha <0.05). *M mode short axis parameters (M)*—LVDd (mm): left ventricular internal diameter (diastole); LVDs (mm): left ventricular internal diameter (systole); LV PWd (mm): left ventricular posterior wall (diastole);LV PWs (mm): left ventricular posterior wall (systole); LVMass (mg): left ventricular mass; LV VOLd (μL): left ventricle volume (diastole); LV VOLs (μL): left ventricle volume (systole). *B mode long axis parameters (B)*—CO (mL/min): left ventricular cardiac output; EF%: ejection fraction; FS%: fractional shortening; SV (μL): stroke volume. *Group numbers*: sedentary female (*n* = 8), RotaHIIT female (*n* = 7), sedentary male (*n* = 7), RotaHIIT male (*n* = 9) for all measures. Bolded value is significantly different compared to pre‐training value (*P*<0.05).

### 3.4 Body composition and skeletal muscle mitochondrial energy production and biogenesis are not altered by RotaHIIT

Previous studies have demonstrated increased skeletal muscle oxidative capacity (Callahan, Parr, Hawley, & Camera, 2021), and anabolic gains in lean muscle mass in parallel with improved aerobic capacity (Callahan, Parr, Snijders, et al., 2021) following HIIT training in humans. To evaluate whole animal changes in tissue composition, we measured body composition through NMR spectroscopy. The average change in lean mass was significantly greater in female RotaHIIT trained versus sedentary mice (*p* < 0.05) (Figure S5). There were no significant differences in percent lean mass found between male RotaHIIT and sedentary mice, or in percent fat mass in RotaHIIT compared to sedentary mice in either of the sexes. We additionally focused specifically on mitochondrial energy production and biogenesis as this molecular mechanism of adaptation has been shown to be activated by high‐intensity training (Bishop et al., 2019; Hood et al., 2016). We performed western blots for each of the five oxidative phosphorylation complexes and mitochondrial transcription factor A (TFAM), which is a key regulator of mitochondrial genome replication, in isolated left ventricular cardiac tissue and gastrocnemius skeletal muscle tissue (Kang et al., 2007; Vina et al., 2009; Zinovkina, 2019). Upregulation of these proteins would indicate increased mitochondrial content (Fiorenza et al., 2019; Popov, 2020; Youssef et al., 2022). Protein lysates from females and male hearts and skeletal muscle were interrogated separately, with fold change being calculated in RotaHIIT trained mice relative to sedentary mice. None of the five oxidative phosphorylation complexes tested were significantly different in cardiac or skeletal muscle tissue in RotaHIIT compared to sedentary mice (Figures S6 and S7). The level of TFAM was also not significantly different in either the left ventricle or gastrocnemius, across both sexes.

## 4 DISCUSSION

We designed a forced exercise training model for mice that takes advantage of the superior adaptive benefits of HIIT (MacInnis & Gibala, 2017), combined with the use of a rotarod that overcomes limitations of treadmill training. The results of our study demonstrate that RotaHIIT elicits a significant training effect on exercise performance, measured by both treadmill and rotarod testing modalities, in both female and male C57BL/6J mice.

Attaining reproducible workload intensities to elicit a training effect from treadmill interval training typically requires weekly GXT testing to ensure an adequate training stimulus over time (Kemi et al., 2002). This can be cumbersome as well as time, labor and resource intensive (Petrosino et al., 2016; Poole et al., 2020). In our experience, GXT testing requires ~1 h per mouse. We adapted workload intensities for RotaHIIT by conducting average weekly maximum acceleration tests on the rotarod for the experimental cohort. The acceleration test allows five mice to be assessed during a single session and takes a period of ~20 min to conduct, including cleaning of the rotarod apparatus. Across the intervention period of 7 weeks, we observed a robust increase in average maximum acceleration capacity in both female and male mice, indicating a substantial training response over time. The resulting group average maximum acceleration capacity was then used for more precise adjustment of weekly training intensities (50% and 80% of maximum for low‐ and high‐intensity intervals, respectively), in order to provide an appropriate training stimulus during each week of the experiment. Female and male mice had significantly different workload intensities during the first two of seven weeks; however, the male mice caught up and there was no difference in subsequent weeks. Therefore, female and male mice could be trained at the same time, which would further lessen experimental time and logistics when conducting training. Notably, as seen in Figure S1, the corresponding linear speeds that the mice achieve on a rotarod (~4 m/min) are significantly slower than the maximum linear speeds achieved during graded exercise testing on a treadmill (~27 m/min, see Supplementary excel data file). This is likely a consequence of the distinct difference between treadmill versus rotarod exercise, including the requirements to maintain balance and overcome body weight resistance at times to remain on the rotating rod. Nonetheless, the RotaHIIT training still translates to improve pVO_2_ during treadmill GXT—the gold standard of exercise capacity—results that support the generalizability of the training model.

Previous reports demonstrate progressively poor compliance with treadmill training in male C57BL/6J mice, with typical completion rates of 50%–60% of sessions during a 4‐week exercise training intervention (Gibb et al., 2016). Low compliance results in lower training volume, and correlates with a lesser or no increase in exercise capacity measured as distance run or work completed (Gibb et al., 2016). Throughout the entirety of the 7‐week RotaHIIT intervention period, both female and male mice averaged greater than 98% compliance of the training sessions. There was no sex difference in compliance during any week of training. During the later weeks of the training protocol, specifically Weeks 6 and 7, we did observe a greater amount of variability in both sexes, owing to the difficulty of the higher training intensities on the rotarod. Future studies using this protocol should account for this variability. An additional advantage of the rotarod is that mice are continuously conditioned (i.e., constantly moving), unlike treadmill training where stop and go running patterns are typical and can limit the overall exercise training response (Poole et al., 2020). The excellent compliance and continuous conditioning of RotaHIIT produced favorable training effects measured by both treadmill GXT testing and rotarod specific exercise capacity tests.

RotaHIIT resulted in significant training effects measured by different testing modalities. Female and male demonstrated increases of 19% and 8% respectively in pVO_2_ measured on a treadmill above sedentary counterparts after 31 min of training/day for 3 days/week over 7 weeks. These pVO_2_ percent increases were lower compared to a treadmill HIIT study conducted by Wisloff et al 2001, which found pVO_2_ increases of 60%–70% in trained female and male adult rats above sedentary controls. The training volume, intensity, and time, however, were much greater than in our protocol—2 h/day, 5 days/week for 7 weeks, with the highest intensity intervals conducted for 8 min at 80%–90% of pVO_2_ (Wisloff et al., 2001). The pVO_2_ training effect in our current study was more comparable to a prior study of endurance treadmill training, in which female and male mice increased pVO_2_ above sedentary controls by 12% and 6%. However, this magnitude of training effect was elicited after a much higher volume of training—2 h of endurance training/day for 6 days/week over 4 weeks (Shefer & Talan, 1997). RotaHIIT produced a similar training effect with much lower cumulative volumes, which is more practical for the purposes of experimentation, reduces stress on the animals, and more closely resembles evidence‐based protocols in patients (Ito, 2019). Of note, we did not observe mean group RER values greater than 1, which is used as anaerobic threshold criteria for assessing maximal oxygen consumption, even though the mice were challenged to the point of exhaustion per the mouse specific GXT (Petrosino et al., 2016). However, RER values did not differ per group, suggesting that no single group was over or under challenged during GXT.

The training effects were greatest when post‐training testing was done using the same modality (rotarod acceleration and endurance capacity) but was still generalizable to the treadmill modality during GXT testing. Our study also determined that the improvements in acceleration and endurance capacity in both male and female mice were significantly correlated with increases in pVO_2_, suggesting that functional capacity on the rotarod could serve as a surrogate for GXT testing on a treadmill. Employing functional capacity testing on a rotarod could save appreciable expenses, time and resources for assessing changes in exercise capacity associated with disease state and/or in response to an intervention and would be a suitable endpoint when pVO_2_ testing is unavailable. One limitation of the RotaHIIT model is that the training did not elicit physiological adaptations in cardiac structure or function that have been demonstrated in other exercise protocols evaluated in healthy murine models (Verboven et al., 2019; Zheng et al., 2022), including increased stroke volume, cardiac output, or left ventricular chamber enlargement and hypertrophy. This observation may be attributable to the relatively lower volume and duration of training compared to prior studies. Therefore, the RotaHIIT model may not be suitable for studies in which physiologic cardiac adaptation to exercise is an important endpoint.

In order to assess underlying mechanistic adaptations, we further interrogated changes in whole body skeletal muscle composition and skeletal muscle oxidative capacity, in addition to oxidative capacity in the heart. HIIT training can increase lean muscle mass, which can contribute to aerobic exercise capacity (Callahan, Parr, Snijders, et al., 2021). We did observe a statistically significant increase in lean body mass in RotaHIIT‐trained female mice and a trend in male mice (Figure S5), suggesting a plausible association between RotaHIIT training and increased capacity for peripheral oxygen extraction.

While mitochondrial biogenesis has been shown to increase with exercise training, we did not observe significant changes in any of the five subunits responsible for oxidative phosphorylation or the mitochondrial genome regulator TFAM. However, these findings do not exclude a peripheral adaptive response, as other factors, such as increased lean muscle mass and angiogenesis (Baek et al., 2022; Kwak et al., 2018) could improve the ability of the skeletal muscle to extract oxygen. Our inability to definitely uncover a mechanism for the training effect produced by RotaHIIT is a limitation of the present study and future studies will be needed to determine the basis for this training effect.

The use of the rotarod for exercise training does not employ an electric shock grid or other aversive stimuli (e.g., air puffs) often needed in treadmill training to motivate running (Poole et al., 2020). Our RotaHIIT protocol was intentionally devoid of stressful stimuli, with only minimal assistance using long forceps to restore transient imbalance during training. No assistance was provided during workload intensity measurements, or during acceleration or endurance capacity testing. Treadmill training also can result in bodily injury due to high speeds, lane barriers or at the junction of the tread and electric shock grid (Poole et al., 2020). No mice were injured on the rotarod, although falls from the rotarod could be stressful and is a potential limitation. However fall distance and impact were mitigated through safety modifications including padding, fall distances of 1–1.5 inches, and operator diligence. The RotaHIIT protocol has yet to be tested in rodent disease models, but it should be recognized that running on a rotarod tests balance and coordination in addition to cardiovascular fitness. Therefore, assessment of functional capacity could be confounded in the setting of neuromuscular pathologies (Bylund et al., 2008), poor motor coordination (Deacon, 2013), or obesity (Herrera et al., 2020). Furthermore, we tested middle aged mice in our study given that functional decline becomes more prominent with age (Justice et al., 2014), and exercise interventions that target middle age cardiovascular comorbidities (e.g., cardiac rehabilitation) are typically employed in middle aged adults (Khera et al., 2017). Future studies in younger and older murine models will provide valuable insights as to the generalizability of RotaHIIT across the age spectrum.

In conclusion, there is growing recognition of the importance of evaluating and modulating exercise and functional capacity in various mouse models of cardiovascular disease. In this context, RotaHIIT will offer an additional option for exercise training that is low risk of injury, low cost, high throughput, and time efficient. The high level of compliance, as well as the consistency and magnitude of training effect at relatively low exercise volumes, make RotaHIIT a valuable alternative or complement to other modes of exercise training in mice.

## AUTHOR CONTRIBUTIONS

Jonathan Herrera conceived and designed research, performed experiments, analyzed data, interpreted results of experiments, prepared figures, drafted manuscript, and approved the final version of the manuscript. Christopher McAllister designed and performed experiments, analyzed data, interpreted results, prepared figures and text, and approved the final version of the manuscript. Danielle Szczesniak and Rose‐Carmel Goddard performed experiments and approved the final version of the manuscript. Sharlene Day conceived and designed research, interpreted results of experiments, edited and revised the manuscript, and approved the final version of the manuscript.

## FUNDING INFORMATION

Jonathan Herrera received T32 support from the University of Michigan Medical Scientist Training Program (MSTP) and Molecular & Integrative Physiology (MIP) programs (GM007863‐38 and GM008322‐28,29), and from the American Physiological Society—the Porter Physiology Predoctoral Fellowship. Sharlene M. Day received support for this project from internal funding mechanisms at the University of Michigan, including the Taubman Scholar Award and the Office of Research Small Scale Grant.

## CONFLICT OF INTEREST STATEMENT

Sharlene Day received consulting fees from Lexicon Pharmaceuticals and Cytokinetics for a data monitoring board. Dr. Day receives grant funding from Lexicon Pharmaceuticals and Bristol Myers Squibb. None of these relationships are relevant to the work presented here.

## ETHICS STATEMENT

All protocols were submitted to, and approved, by the University of Michigan Institutional Animal Care and Use Committee.

## Supporting information


Data S1.

Figure S1.

Figure S2.

Figure S3.

Figure S4.

Figure S5.

Figure S6.

Figure S7.

 


## Data Availability

The data are available on request from the authors.
