# Supplementary figures and images for: High‐intensity exercise training using a rotarod instrument (RotaHIIT) significantly improves exercise capacity in mice
Source: Physiol Rep. 2024 May 2;12(9):e15997. doi: 10.14814/phy2.15997 (PMC11065697; doi:10.14814/phy2.15997)

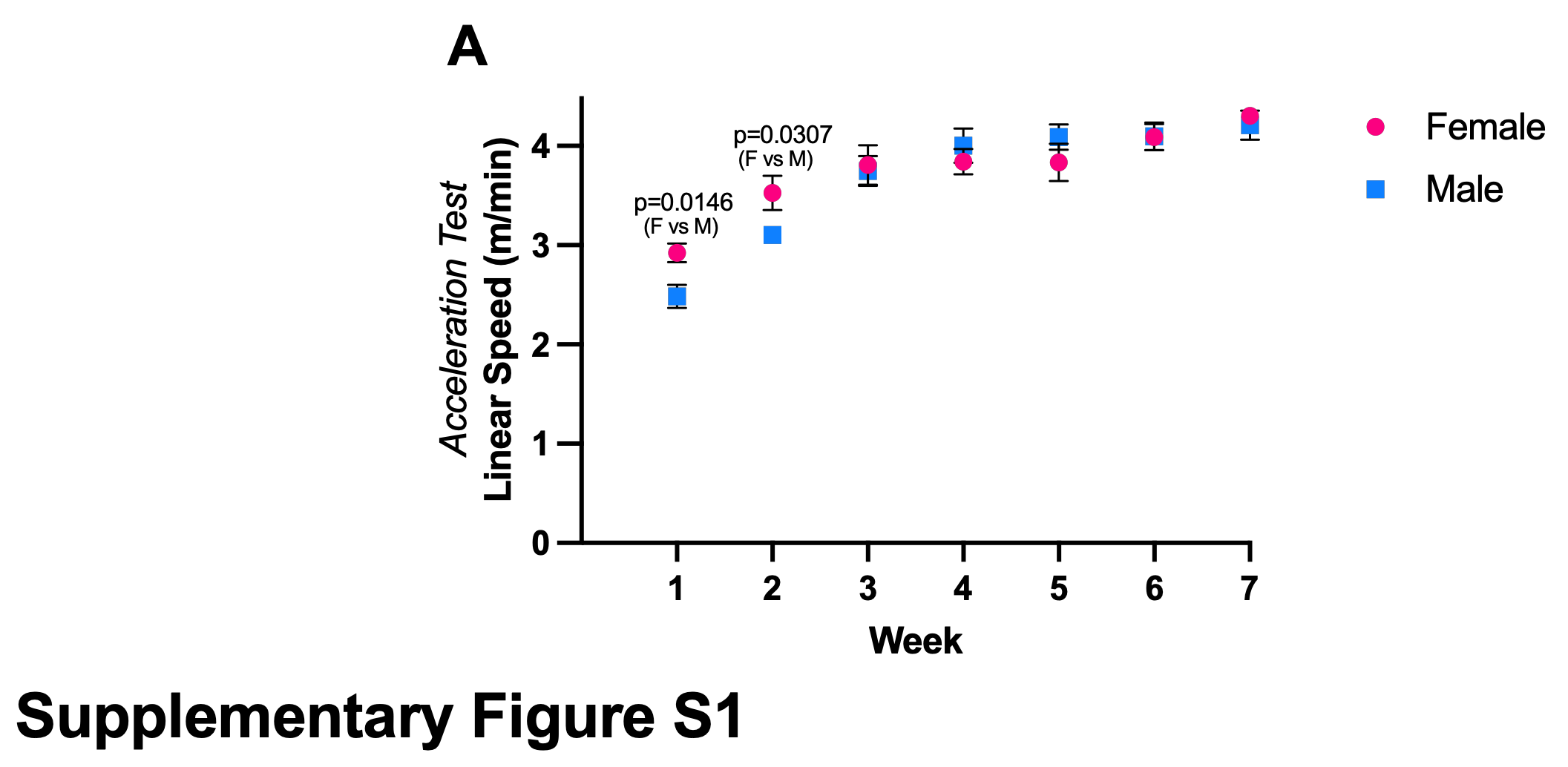

Supplement: Supplementary file 2 — Figure S1. [file PHY2-12-e15997-s004.tif]

**A**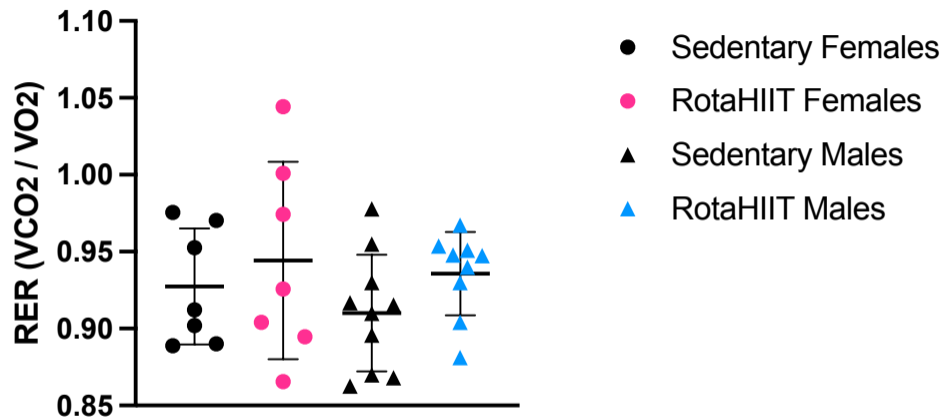

**Supplementary Figure S2**

Supplement: Supplementary file 3 — Figure S2. [file PHY2-12-e15997-s008.pdf]

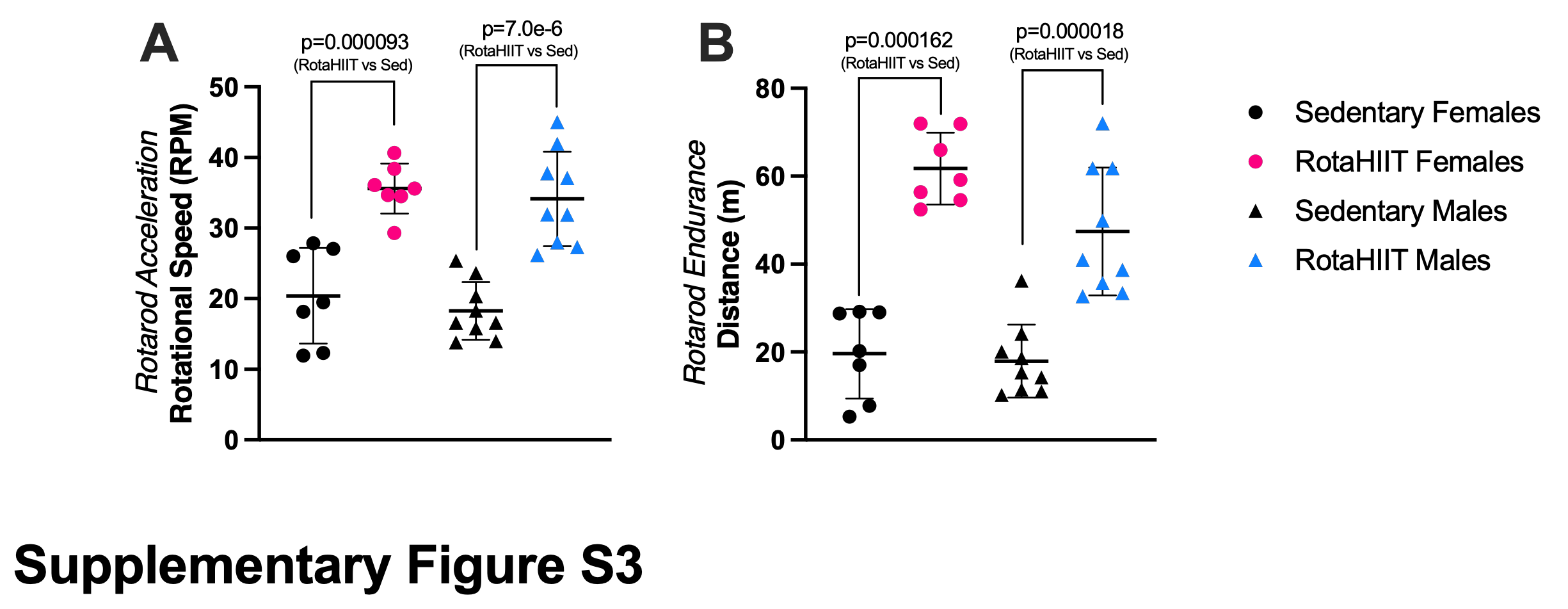

Supplement: Supplementary file 4 — Figure S3. [file PHY2-12-e15997-s002.tif]

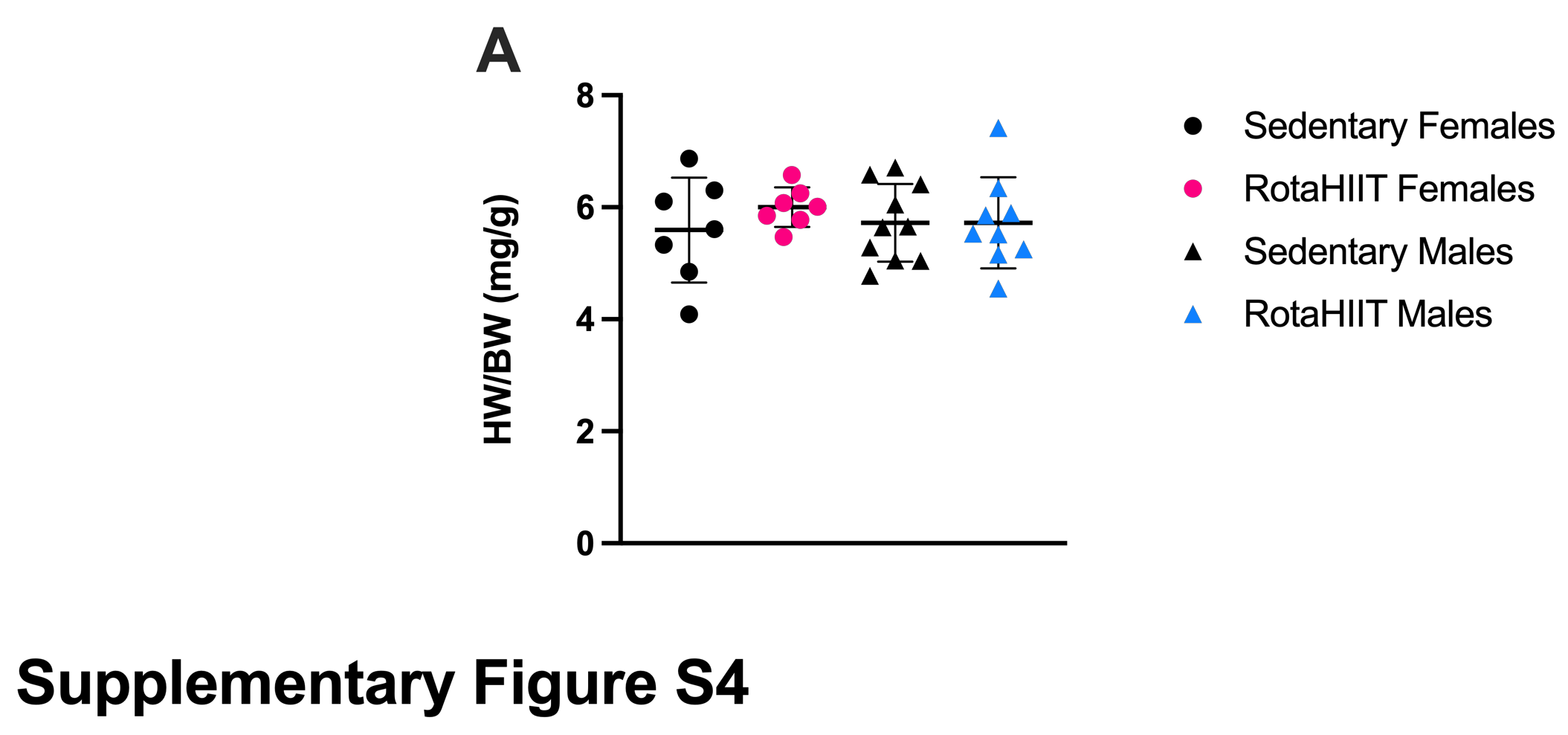

Supplement: Supplementary file 5 — Figure S4. [file PHY2-12-e15997-s005.tif]

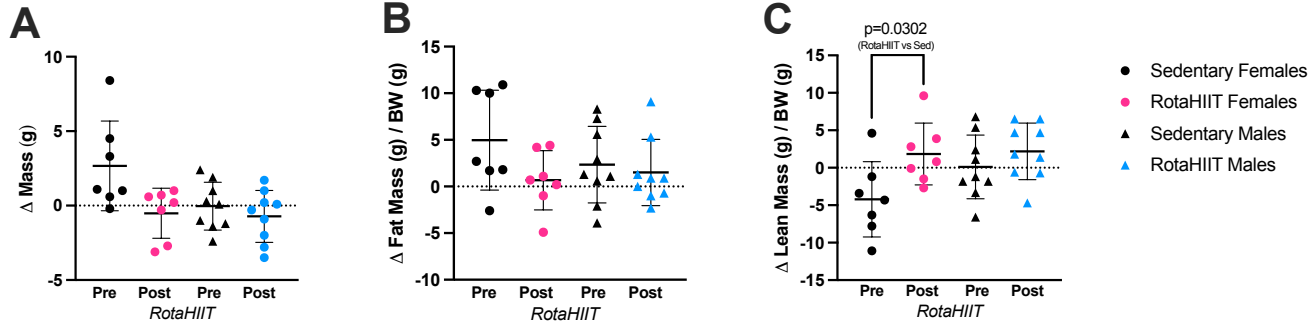

**Supplementary Figure S5**

Supplement: Supplementary file 6 — Figure S5. [file PHY2-12-e15997-s006.pdf]

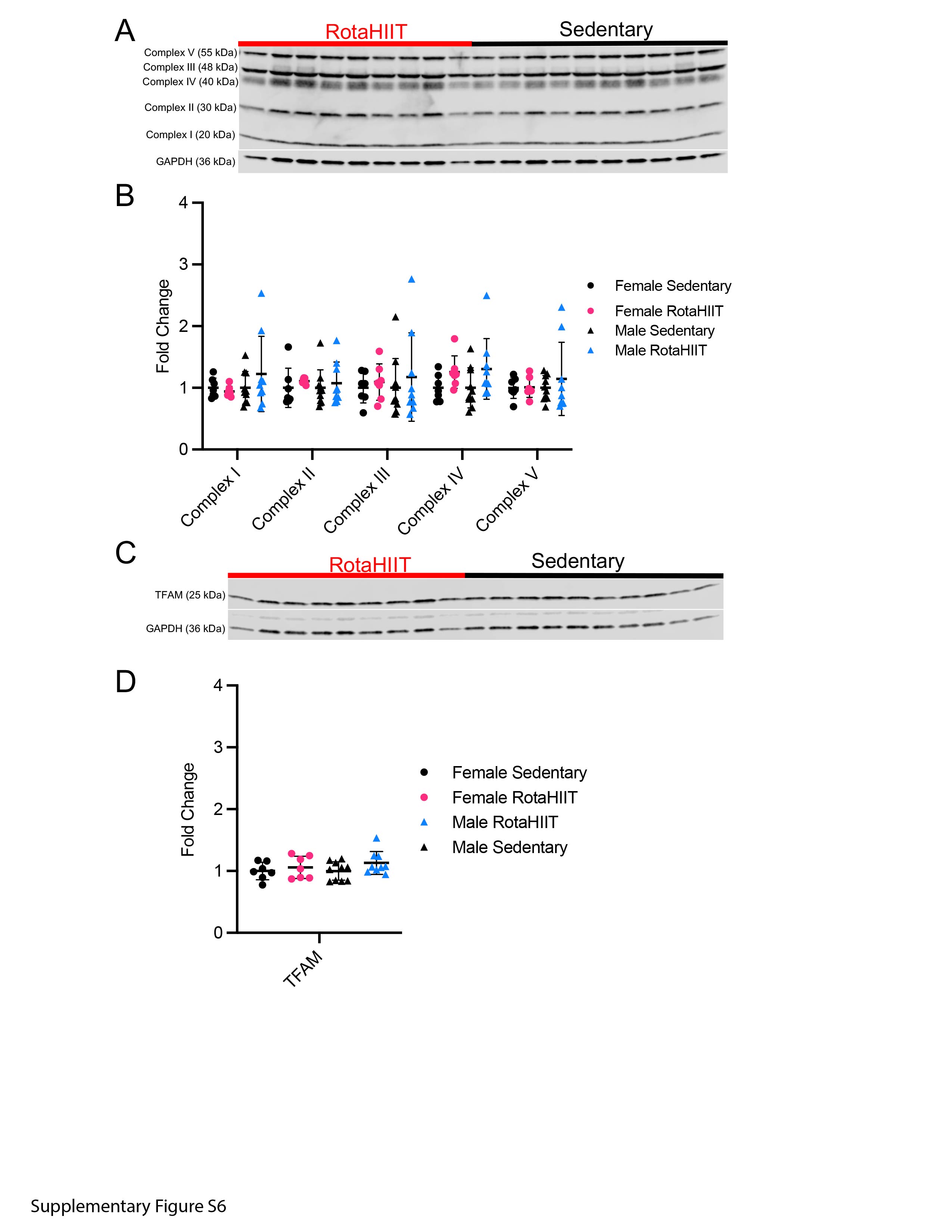

Supplement: Supplementary file 7 — Figure S6. [file PHY2-12-e15997-s003.jpg]

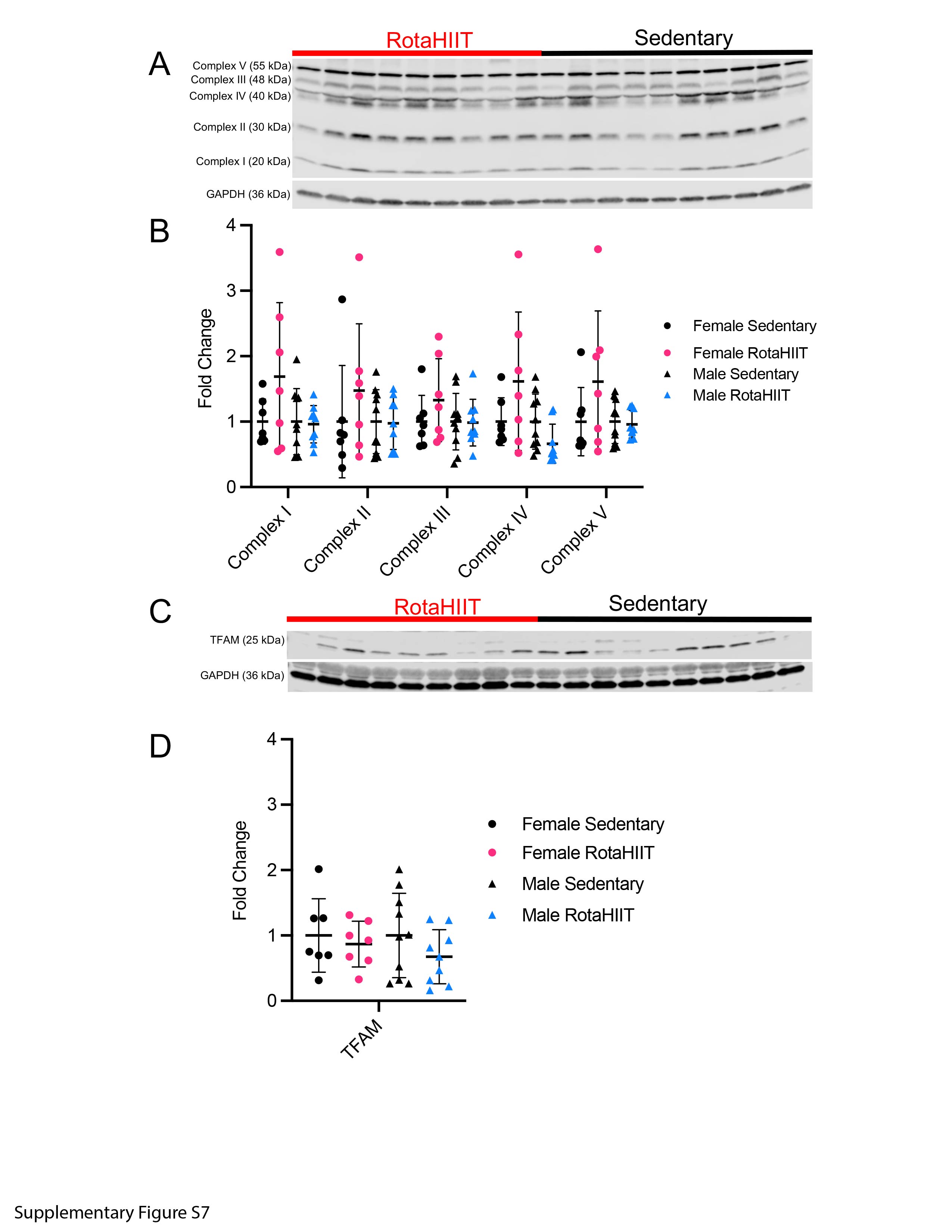

Supplement: Supplementary file 8 — Figure S7. [file PHY2-12-e15997-s001.jpg]
