# Supplementary material for: High‐intensity exercise training using a rotarod instrument (RotaHIIT) significantly improves exercise capacity in mice
Source: Physiol Rep. 2024 May 2;12(9):e15997. doi: 10.14814/phy2.15997 (PMC11065697; doi:10.14814/phy2.15997)
Supplement: Supplementary file 9 [file PHY2-12-e15997-s007.docx]

**PHY215997**

**Supplementary figure legends:**

**Supplementary Figure S1.** **Average weekly maximum acceleration (converted from rotational speed to linear velocity).** Prior to the start of each week of exercise training, mice were subjected to a rotarod acceleration capacity test to establish RotaHIIT workloads. (A) Average maximum acceleration in meters/min (m/min) (± standard error) for female and male mice are plotted for each week (Note: Week 1 was utilized an as acclimation period for mice to become accustomed to rotarod). Unpaired T-test was used to compare female and male averages per week (alpha threshold, p < 0.05).

**Supplementary Figure S2.** **Mean respiratory exchange ratio (RER) during post graded exercise testing (GXT).** After 6 weeks of RotaHIIT, graded exercise testing (GXT) was conducted in a metabolic treadmill chamber allowing for volume of oxygen consumed (VO_2_) and volume of carbon dioxide produced (VCO_2_) by mice to be measured. (A) The average RER per group achieved prior to exhaustion is plotted as the ratio of VCO_2_ to VO_2_. One-way ANOVA was used to compare changes across groups. Tukey’s post-hoc test was used to determine individual comparisons with alpha threshold of p <0.05.

**Supplementary Figure S3.** **Rotarod post-RotaHIIT exercise capacity measures conversion.** Following 6 weeks of RotaHIIT training, mice were assessed for exercise capacity using Rotarod specific tests. (A) Average maximum rotational speed in revolutions per minute (RPM) achieved when rotarod was continuously accelerated at rate of 0.1 RPM/sec (ie, Acceleration Capacity). (B) Average total distance in meters achieved when rotational speed was maintained at fixed speed (31 RPM). One-way ANOVA was used to compare changes across groups. Tukey’s post-hoc test was used to determine individual comparisons with alpha threshold of p <0.05.

**Supplementary Figure S4.** **Heart weight normalized to body weight.** Following 6 weeks of RotaHIIT training, hearts were extracted and weighed. (A) Average heart weights of experimental groups normalized to post mortem body weight. One-way ANOVA was used to compare changes across groups. Tukey’s post-hoc test was used to determine individual comparisons with alpha threshold of p <0.05.

**Supplementary Figure S5.** **Change in body mass and composition following RotaHIIT.** Measurements of body mass and body composition were made prior to and following RotaHIIT training. Each points plotted value represents post intervention minus pre intervention for a single mouse. (A) Body mass was measured using animal weighing scale. Fat mass (B) and lean mass (C) were measured using an NMR based analyzer. One-way ANOVA was used to compare changes across groups. Tukey’s post-hoc test was used to determine individual comparisons with alpha threshold of p <0.05. Repeated measures T-test was performed to assess mean differences within each group with alpha threshold of p<0.05.

**Supplementary Figure S6. Mitochondrial biogenesis and oxidative phosphorylation components in the heart.** (A-B) Representative western blot and quantification for oxidative phosphorylation complexes I-V in tissue isolated from the mouse left ventricle (n=6-10). (C-D) Representative western blot and quantification for mitochondrial transcription factor A (TFAM) in tissue isolated from the mouse left ventricle (n=6-10). Analysis was performed on hearts from both male and female mice independently following completion of RotaHIIT. Data are expressed as mean ± SD. Protein abundance was normalized to GAPDH, and fold change was calculated relative to the sedentary condition. Unpaired t test with a threshold of p<0.05 was used to determine statical significance.

**Supplementary Figure S7. Mitochondrial biogenesis and oxidative phosphorylation components in the skeletal muscle.** (A-B) Representative western blot and quantification for oxidative phosphorylation complexes I-V in tissue isolated from the gastrocnemius muscle (n=6-10). (C-D) Representative western blot and quantification for mitochondrial transcription factor A (TFAM) in tissue isolated from the gastrocnemius muscle (n=6-10). Analysis was performed on the skeletal muscle of both male and female mice independently following completion of RotaHIIT. Data are expressed as mean ± SD. Protein abundance was normalized to GAPDH, and fold change was calculated relative to the sedentary condition. Unpaired t test with a threshold of p<0.05 was used to determine statical significance.
